# Anthropogenic Intensity-Determined Assembly and Network Stability of Bacterioplankton Communities in the Le’an River

**DOI:** 10.3389/fmicb.2022.806036

**Published:** 2022-05-04

**Authors:** Bobo Wu, Peng Wang, Adam Thomas Devlin, Yuanyang She, Jun Zhao, Yang Xia, Yi Huang, Lu Chen, Hua Zhang, Minghua Nie, Mingjun Ding

**Affiliations:** ^1^School of Geography and Environment, Jiangxi Normal University, Nanchang, China; ^2^Key Laboratory of Poyang Lake Wetland and Watershed Research, Ministry of Education, Jiangxi Normal University, Nanchang, China; ^3^School of Geography and Ocean Science, Nanjing University, Nanjing, China

**Keywords:** human activity intensity, assembly processes, bacterioplankton, network stability, river

## Abstract

Bacterioplankton are essential components of riverine ecosystems. However, the mechanisms (deterministic or stochastic processes) and co-occurrence networks by which these communities respond to anthropogenic disturbances are not well understood. Here, we integrated niche-neutrality dynamic balancing and co-occurrence network analysis to investigate the dispersal dynamics of bacterioplankton communities along human activity intensity gradients. Results showed that the lower reaches (where intensity of human activity is high) had an increased composition of bacterioplankton communities which induced strong increases in bacterioplankton diversity. Human activity intensity changes influenced bacterioplankton community assembly *via* regulation of the deterministic-stochastic balance, with deterministic processes more important as human activity increases. Bacterioplankton molecular ecological network stability and robustness were higher on average in the upper reaches (where there is lower intensity of human activity), but a human activity intensity increase of about 10%/10% can reduce co-occurrence network stability of bacterioplankton communities by an average of 0.62%/0.42% in the dry and wet season, respectively. In addition, water chemistry (especially NO_3_^–^-N and Cl^–^) contributed more to explaining community assembly (especially the composition) than geographic distance and land use in the dry season, while the bacterioplankton community (especially the bacterioplankton network) was more influenced by distance (especially the length of rivers and dendritic streams) and land use (especially forest regions) in the wet season. Our research provides a new perspective of community assembly in rivers and important insights into future research on environmental monitoring and classified management of aquatic ecosystems under the influence of human activity.

## Introduction

Human activity refers to the process of anthropogenic utilization, exploitation, and protection of the natural environment for development and survival (Xu et al., 2015). Changes in water quality caused by the alteration of water supply, drainage, and the construction of impervious surface (Yang et al., 2022) can lead to changes of environment conditions and land use, and an alteration of aquatic ecosystems in proximity to urban areas (Uchida et al., 2020). Rivers are critical freshwater ecosystems that interact with other ecosystems through constant input from surface runoff (Zhang L. et al., 2020; Li et al., 2021). Bacterioplankton are key components of freshwater ecosystems which compose a significant portion of the biodiversity and play a significant role in biogeochemical cycles (Wang Y. et al., 2020). Additionally, bacterioplankton diversity is an important indicator of water quality (Wang B. et al., 2020). Understanding bacterioplankton community parameters (e.g., structure, composition, and distribution) is critical in accurately determining the mechanisms which drive microbial community assembly. Riverine ecosystems can be severely influenced by human-related activities which can lead to changes in species diversity, community composition, and biotic integrity at different trophic levels (Vishnivetskaya et al., 2011; Ouyang et al., 2020; Wu B. et al., 2021). In particular, changes in land use can have dramatic effects on riverine microbial ecosystems (Li et al., 2020). The spatial distributions of bacterioplankton communities can be affected by anthropogenic land use urbanization, industrialization, and agriculturalization (Zhao et al., 2021). In addition, changes in riparian land cover or flow alteration of flow damming (e.g., dams) can impact organic matter retention in rivers, filter keystone species, reshape distribution of metacommunities, mediate ecological assembly processes of microbial communities, and subsequently alter the stability of ecosystem processes (Grill et al., 2019; Wang Y. et al., 2020; Gao et al., 2021). Therefore, a better understanding of river bacterioplankton ecology requires exploration of the composition and network of bacterioplankton communities in riverine ecosystems. However, a comprehensive understanding of how human activity intensity impacts river biodiversity and the bacterioplankton network is still lacking.

Exploring community assembly mechanisms of bacterioplankton and their related ecological processes is paramount in the field of community ecology (Nemergut et al., 2013). Deterministic (Niche-based) and stochastic (neutral-based) processes can both explain the assembly and community structure of bacterioplankton (Bahram et al., 2016). The deterministic process asserts that bacterioplankton communities are shaped by biotic and abiotic factors, such as species interactions (e.g., predation and competition) and environmental filtering (e.g., temperature and pH) (Liu et al., 2015). The stochastic process asserts that processes such as random birth, death, speciation, immigration, and extinction shape the bacterioplankton community structure (Vanwonterghem et al., 2014). Determinism and stochasticity have diverse effects on bacterioplankton communities in coastal regions (Mo et al., 2018), rivers (Sun et al., 2021), and lakes and reservoirs (Liu et al., 2015). Significant deterministic relationships between bacterioplankton composition and environmental parameters have been previously reported for multiple spatial scales (Agogué et al., 2010; Glaeser et al., 2010; Shu et al., 2020). However, stochastic processes also can explain the bacterioplankton community composition in diverse aquatic environments at large geographical scales (Zhao et al., 2017; Chen et al., 2019; Logares et al., 2020). Therefore, both approaches have utility, and no overall consensus has yet been found. Given that human activity changes may be related to environmental filtering, understanding key ecological processes that govern bacterioplankton community assembly in rivers subject to strong anthropogenic influences is critical for sustainable watershed management.

Future increases of anthropogenic activity will continue to disrupt natural environments, increase environmental stress, and amplify the destabilization of microbial co-occurrence networks (Shu et al., 2021). Bacterioplankton species in natural ecosystems do not exist in isolation or appear in individual populations (Mo et al., 2021). These species interact to form complex bacterioplankton communities and perform multiple ecosystem functions (Jiao C. et al., 2020). Thus, the understanding of bacterioplankton communities is best focused on individual species level characteristics (e.g., species abundance and richness) as well as the interaction characteristics of the communities. Most bacterioplankton taxa interact either directly or indirectly, and either positively, negatively, or neutrally with each other through multiple mechanisms to form complex ecological networks and drive ecosystem functionality (Jiao C. et al., 2020). The community response to human disturbances can be impacted by ecological network properties (Hernandez et al., 2021). For example, negative interactions and network modulations can enhance network stability (Coyte et al., 2015). Although there exists an extensive body of novel and innovative network analysis methods in ecology (Jiao C. et al., 2020; Xue et al., 2020; Shu et al., 2021), there is still much work needed to better understand how the network stability of bacterioplankton communities will respond to various human activity intensities. Co-occurrence network analysis can be utilized to understand the network structure of bacterioplankton and its environmental drivers in rivers.

The Le’an River, a mid-sized river in subtropical China, is a suitable system to study the effect of strong anthropogenic disturbances on community assembly processes (Yu et al., 2016). The Le’an River is under severe environmental stress due to mining occurring near the middle reaches of the river, nutrition pollution from domestic and agricultural operations in the downstream reaches, and other anthropogenic influences (Chen et al., 2016; Ji et al., 2018; Zhang H. et al., 2020). However, the relationships between topological structures of bacterioplankton networks of the Le’an River and how the anthropogenic activity has impacted niche-neutrality successional mechanisms in a freshwater continuum is yet an open question. Thus, the present study has three main objectives: (1) explore how the deterministic processes and stochastic processes cooperate with each other in bacterioplankton community assemblage; (2) reveal the differences and the interaction mechanisms of bacterioplankton community networks in response to spatially varying human activity intensity and (3) identify the relative influences of land use, water chemistry, and geographic distance in bacterioplankton communities (e.g., composition and network).

## Materials and Methods

### Study Area and Sampling Methodology

**FIGURE 1:**
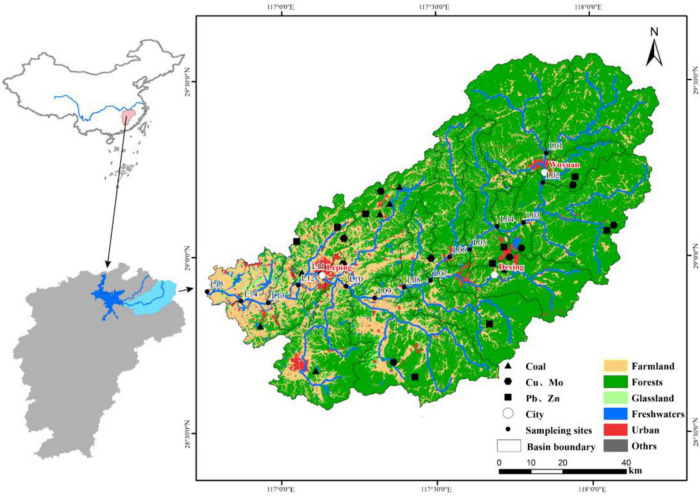
Location of the Le’an River and our sample sites. The cities of Wuyuan, Dexing, and Leping are indicated.

The Le’an River course goes through multiple counties (Wuyuan, Dexing, and Leping), empties into Poyang Lake which then goes into the Chang (Yangtze) River (Figure 1). The Le’an River is ∼ 279 km long and has a drainage area of 8989 km^2^. The basin is situated in a monsoon climate zone, with an average annual precipitation of 1850 mm. There is a long wet season from April to September, with 69.1% of the annual precipitation falling during this period. The Le’an River basin has many non-ferrous metal mines, including the most productive copper mine in China (Dexing), located in the middle reaches of the river. The downstream areas are surrounded by rice cropping agriculture.

Water samples were collected at an approximate depth of 50 cm at 15 sites (Figure 1) located in the center of the river channel. We attached organic glass hydrophore to a long rope, stood on the bridge, and carefully dropped it into the river water below, waiting for 5 min for the water to saturate the sampler, and then pulling it back. Samples were collected in both dry season and wet season periods (December 2016 and June 2017, respectively). Sites L01–L04 are in the upper reaches, sites L05–L10 are in the middle reaches, and sites L11–L15 are in the lower reaches. We used several (4–6) filtering equipment with several hand pumps at once for one water sample (3 L). Then all filters from one sample were mixed together for environmental DNA extraction. In addition, we use the prefiltration to reduce the filtering time. The procedure of prefiltration for water sample is recommended by the Shanghai Majorbio Bio-pharm Technology Co., Ltd., China, where the sequencing was performed. This prefiltration procedure has been used in some studies (Staley et al., 2013; Read et al., 2015; Hu et al., 2017). Water samples were filtered through a 0.45 μm acetate filter membrane, then placed into a sealed sampling bottle and refrigerated at 0–4°C. Electric conductivity (EC) and pH tests were carried out using a portable water quality analyzer (HI9828, Hanna Instruments Ltd., Rome, Italy) which was calibrated before each measurement. The calibration of the conductivity/pH meter was performed with buffer and a conductivity solution. The estimated precision was 1 and 1% for pH and EC, respectively. Additionally, an automatic discontinuous analyzer (Smartchem 200 Brookfield, WI, United States) was used to determine ammonia nitrogen (NH4^+^-N), nitrate (NO_3_^–^-N) and total phosphorus (TP). Chlorine and sulfate ion (Cl^–^, SO_4_^2–^) concentrations were determined using an Ics-2100 ion chromatography system. Total organic carbon (TOC) was also measured using a TOC analyzer (Shimadzu TOC-L CPH, Kyoto, Japan). Trace metals, including chromium (Cr); copper (Cu); zinc (Zn); cadmium (Cd); iron (Fe); cobalt (Co); arsenic (As), and lead (Pb) were measured with ICP–MS (Thermo X series II, NE, United States). The accuracy and precision of the methods and results were checked by using the certified Standard Reference Materials (SRM-1640 and SRM-1643e of National Institute of Scientific and Technology, United States). The detection limits were 0.031 (Cr), 1.929 (Fe), 0.005 (Co), 0.091 (Cu), 0.025 (Zn), 0.1 (As), 0.001 (Cd), and 0.026 (Pb).

We employ digital elevation model (DEM) data (∼ 30 m resolution) to delineate the boundaries of the river basin. Sub-basin classifications at each sampling site ranged from a single sampling site that encompassed the sub-basin area to the inclusion of adjacent upper sample sites to reflect the inputs of allochthonous bacteria due to the fast population growth and replacement rates of bacterial communities. Land-cover classification was generated using Landsat 8 satellite imagery (also at 30 m resolution). Land use patterns were determined using imagery from the National Earth System Science Data Center at the National Science and Technology Infrastructure of China^1^. Images were then categorized into six classes: farmlands, forests, grassland, freshwaters, urban areas, and others.” Calculations were performed using ArcGIS v10.3.

We calculate four geographic distance parameters (river length, catchment area, cumulative dendritic distance, and mean dendritic stream length). Here, river length is defined at that found in the mainstream of the river, where the majority of anthropogenic land use types (e.g., towns, cities, and industries) are observed. Thus, river length is the most important distance parameter to analyze human activity. Catchment area can suggest runoff volume. Cumulative dendritic distance is taken as the length of the river networks upstream of a sample site. Finally, mean dendritic stream length is calculated as the ratio of the dendritic stream length (cumulative) to the number of paths from all springs to each sample site. All geographic determinations were performed in ArcGIS v10.3.

### Environmental DNA Extraction, Amplification, and Sequencing

We pre-filtered samples using 5 μm Durapore membrane filters (diameter 25 mm; Xinya, China); this helps to remove particulates and algal biomass. Next, we filter again using 0.22 μm Durapore membrane filters (diameter 25 mm; Xinya, China) to collect microbial cells. Each water sample was filtered simultaneously using multiple membranes to reduce filtering time. Filtered samples were then mixed and stored at −80°C before environmental DNA extraction.

Total DNA from the water samples were extracted using the E.Z.N.A.^>^ Soil DNA Kit (Omega Bio-tek, Norcross, GA, United States), following the manufacturer’s protocols. The bacterial V3–V4 hypervariable regions of 16S rRNA genes were amplified using a forward primer of: 338F (5′-ACTCCTACGGGAGGCAGCA-3′) and a reverse primer of: 806R (5′-GGACTACHVGGGTWTCTAAT-3′) (Castrillo et al., 2017). The V3–V4 hypervariable region has been targeted *via* the MiSeq platform (which can produce single-end reads of 350 bp), which can allow for more accurate and cost-effective characterizations of microbiome samples (Caporaso et al., 2012; Loman et al., 2012; Fouhy et al., 2016). PCRs were then carried out using the following parameters: initial denaturation for 2 min at 95°C, followed by 25 cycles of 30 s at 95°C, annealing for 30 s at 55°C, and elongation for 30 s at 72°C. These were all followed by a final elongation step for 5 min at 72°C. We then use gel electrophoresis on 2% agarose gels to amplify PCR. Triplicate PCR amplicon products were pooled for each sample, then purified using an AxyPrep DNA gel extraction kit (Axygen, Corning, NY, United States), and finally quantified using the QuantiFluor™-ST system (Promega, Madison, Wi, United States). DNA sequencing was conducted on the Illumina MiSeq platform (Illumina, San Diego, CA, United States) and pair-ended using 2 bp × 250 bp sequencing chemistry. All PCR products were sequenced using the Illumina MiSeq platform by Shanghai Majorbio Bio-pharm Technology Co., Ltd., China. Raw reads were deposited in the NCBI Sequence Read Archive database (Accession number: SRP142494).

### Statistical Analyses

Human activity intensity of the land surface can be estimated by the determination of land use/cover types; direct human interference types include farmland and urban use (Xu et al., 2015). The human activity intensity of land surface is given by:


$$\text{HAILS}=\frac{S\,i}{S}\times 100\%$$

where HAILS is the human activity intensity of land surface; *Si* is the human activity-influenced land area; *i* is cover type, and *S* is total land area. Changes in HAILS were calculated and are displayed in Supplementary Table 1. The HAILS level in the Le’an River watershed has a gradient from upstream to downstream: (1) Low HAILS level were L01-L04 sites classified to the upper reaches; (2) Middle HAILS level were L05-L10 sites classified to the middle reaches; (3) High HAILS level were L11-L15 sites classified to the lower reaches.

Raw fastq files were demultiplexed and quality-filtered using QIIME v1.30 as follows: 250 bp reads were truncated at any site with an average quality score b20 over a 50 bp sliding window, and truncated reads shorter than 50 bp were discarded; reads with N2 nucleotide mismatches during primer matching or with ambiguous characters were removed; only sequences that overlapped by N10 bp were assembled on the overlap sequence. Reads that could not be assembled were discarded. We used Usearch v7.0 to identify and remove chimeric sequences, and to cluster OTUs at a 97% similarity cutoff. Operational Taxonomic Units (OTUs) were clustered using a 97% similarity cutoff (UPARSE version 7.1). Chimeric sequences were then identified and removed using UCHIME. The phylogenetic affiliation of each 16S rRNA gene sequence was then analyzed using the RDP Classifier (Release11.3) and compared against the Silva (Release 119) 16S rRNA database employing a confidence threshold of 70%. The OTU taxonomy was assigned against the Newton freshwater 16S rRNA database (Newton et al., 2011) with a 70% confidence threshold by the RDP Classifier (release 11.3^2^) (Wang et al., 2007). Dilution curve analysis was subsequently performed based on OTU values. The Chao1 richness index and the Shannon diversity index were used to assess the alpha diversity. Principal coordinate analysis (PCoA) was then performed on the OTU data which used Bray-Curtis distance matrices to examine similarity among bacterial communities at every site. Similarity analysis (ANOSIM) tests were used to determine if the differences in OTUs among groups were statistically significant. We evaluated clustering or overdispersion of bacterioplankton communities through an examination of the deviation of each observed metric from the average of the null model (checkerboard or C score) (Stone and Roberts, 1990; Gotelli and McCabe, 2002; Crump et al., 2009; Mo et al., 2021). The C-score was evaluated using 30,000 simulations and sequential swap randomization algorithms with the EcoSimR” package in R, version 3.6.1 (R Core Team, 2015). The contribution of stochastic processes to bacterioplankton communities was then estimated using a neutral community model (Sloan et al., 2006). We calculated Levins’ niche breadth (B) index using the formula:


$$B_{j}=\frac{1}{\sum_{i=1}^{N}P_{i\,j}^{2}}$$

*B*_*j*_ is the habitat niche breadth of OTU *j* in each metacommunity, *N* is the total number of communities in each metacommunity and *P*_*ij*_ is the proportion of OTU *j* in community *i* (Pandit et al., 2009). These calculations were performed using the “niche.width” function in R package “spaa” (Pandit et al., 2009; Zhang, 2020).

We then construct Bacterioplankton molecular ecological networks using 16S rRNA and molecular ecological network methods (Deng et al., 2012; Jiao S. et al., 2020; Wu B. et al., 2021). Phyla-level network analysis was performed to identify the relations between microbial taxa using Cytoscape version 3.4.0 combined with the CONET plug-in^3^ (Jiao S. et al., 2020; Xia et al., 2020; Mishra et al., 2021). The topological roles of different nodes were separated into four sub-categories according to their within-module degree (*z*_*i*_) and participation coefficient (*p*_*i*_) threshold value: network hubs (*z*_*i*_ > 2.5; *p*_*i*_ > 0.62), module hubs (*z*_*i*_ > 2.5; *p*_*i*_ < 0.62), connectors (*z*_*i*_ < 2.5; *p*_*i*_ > 0.62) and peripherals (*z*_*i*_ < 2.5; *p*_*i*_ < 0.62) (Poudel et al., 2016; Fan et al., 2018). The “bioenv” function in the “vegan” package was then used to identify environmental factors subsets that best predicted the differences in microbial community structure, which were then used in variance partitioning analysis (VPA) modeling (Borcard et al., 1992; Oksanen, 2012) to evaluate relative contributions of land use, water chemistry, and geographic distance parameters (Supplementary Figure 6). Mantel’s correlations between taxonomic compositions and environmental factors were computed in R (Sunagawa et al., 2015), and all data were tested for normality (Shapiro–Wilkes test). Variables that were not normally distributed were then log transformed to normality. One-way analyses of variance (ANOVA) tests using Fisher’s least significant difference (LSD) *post-hoc* tests were performed in SPSS Statistics v20, at a significance level of *p* ≤ 0.05.

## Results

### Change of Human Activity Intensity and Water Chemistry Characteristics Along the Le’an River

We examine the spatial distribution of farmlands, forests, grassland, freshwaters, urban areas, and others, to understand the impacts of anthropogenic disturbance. Forest areas occupy the largest proportion of the upper reaches of the watershed (84.69 ± 3.37%), whereas a higher percentage of urbanized area is found in the urbanized middle reaches (4.93 ± 2.76%) and a higher percentage of agricultural land is found in the lower reaches (48.60 ± 17.14%).

The Cl^–^ levels in the Le’an River showed a statistically significant differences (*P* < 0.05) between the three HAILS levels. The levels of the NO_3_^–^N, NH_4_^+^-N, Cr, Cu, Zn, Cd, Co and Pb were significantly greater in the dry season than in the wet season (Supplementary Table 2 and Supplementary Figure 1). EC, SO_4_^2–^ and most trace metals levels were at a peak at sites L05–L06 or L09–L12. NO_3_^–^-N and TP concentrations increased from the upper reaches to the lower reaches, and the Cl^–^ was larger in the lower reaches. Higher nutrient levels (mainly N and P) observed in middle and downstream waters are likely associated with strong anthropogenic activities and a higher percentage of urbanized regions in the Le’an River watershed. The presence of extensive mining (e.g., Dexing Copper Mine) leads to the concentrations of Cr, Cu, Zn, and As being highest in the middle reaches. Compared with the surface water environmental quality standards in China (GB3838–2002), the concentrations of NH_4_^+^-N, TP and Pb exceeded the water quality standard values in several samples (Supplementary Figure 1). The result of the *T*-test showed that the concentrations of NO_3_^–^-N, NH_4_^+^-N, Cu, Zn, Cd, and Pb in the dry season were significantly higher than those in the wet season.

The results of RDA showed that the land use and geographic distance together explained 85.87 and 86.19% of the water-quality variations (Supplementary Figure 2). In the wet season, the Mean dendritic stream length (*P* = 0.022) was the most significant variables affecting water quality. In the dry season, the Urban (*P* = 0.041) was the most significant variable affecting water quality.

### Bacterioplankton Community Diversity and Composition Along HAILS Gradient

After filtering for quality and subsampling (24,343 reads per sample), 770 OTUs were acquired with 212–416 per sample. All OTUs were assigned to 478 species, 310 genera, 188 families, 110 orders, 64 classes, and 30 phyla. The number of OTU species in wet and dry seasons and the number of species obtained at various taxonomic levels varied significantly (Supplementary Table 3). Good’s coverage for the observed OTUs was 99.73 ± 0.05%, which suggests a near-complete community sampling.

Both Shannon diversity and Chao1 richness indices for the bacterioplankton community were significantly higher in the wet season than in the dry season, and both slightly increased in fluctuations from the upper reaches to the lower reaches (Supplementary Figure 3). Three distinct clusters are observed in the PCoA plots of the bacterioplankton community in the dry season and the wet season, distributed in an orderly manner from upper reaches to middle reaches (Supplementary Figure 4). ANOSIM tests gave a global R value of 0.515/0.611 at *p* = 0.002/0.001, indicating statistically significant separation of the two seasons. Community clustering could explain more of the observed variation in the wet season than in the dry season. Furthermore, the difference of seasonal variations of the bacterioplankton community composition was not statistically significant, as indicated by the ANOSIM (*P* = 0.14) tests. Therefore, geographic distance between the three reaches had greater impact on bacterioplankton community composition than season.

**FIGURE 2:**
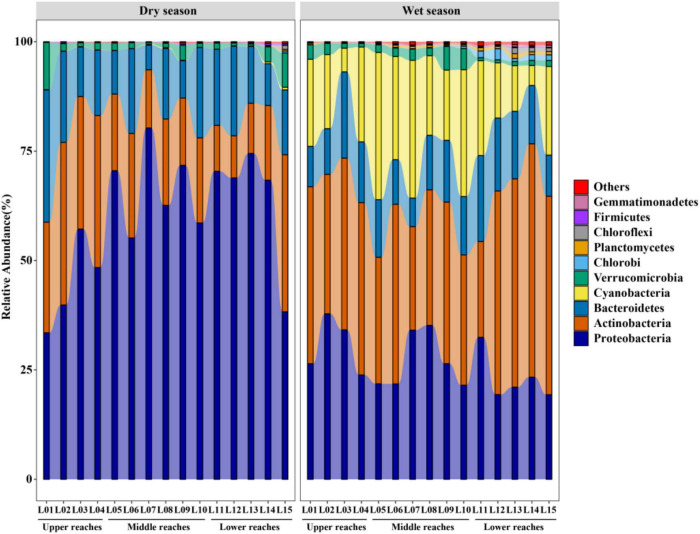
Relative bacterioplankton community abundances of during the wet and dry seasons, from upstream to downstream. Compositions are shown as bacterioplankton phyla distributions. Taxa with relative abundances <1% are grouped in red and labeled as “others.”

The most common phyla observed, in order of occurrence, were *Proteobacteria*, *Bacteroidetes*, and *Actinobacteria* (Figure 2). *Proteobacteria* (59.9%) were the large majority, and were much more abundant in the dry season than in the wet season. *Proteobacteria* abundance was significantly lower in the upper reaches as compared to the lower and middle reaches during the dry season. *Proteobacteria* abundance differed at different sampling sites in the wet season. *Actinobacteria* was the most abundant during the wet season (37.1%), much more than in the dry season (21.4%). The abundance of *Actinobacteria* was significantly lower in the middle reaches in both the dry and wet seasons. *Bacteroidetes* did not significantly differ between the dry season (15.5%) and the wet season (13.2%). *Bacteroidetes* abundance was stable at all sites in both seasons. In contrast, *Cyanobacteria* were the third most abundant phylum in the wet season (19.0%), but had very low abundance during the dry season (0.1%). In the lower reaches, *Cyanobacteria* abundance (13.9%) were significantly lower in the wet season, as compared to the middle reaches (25.3%). In addition, RDA was used to determine the relationship between the main bacterioplankton phylum and environmental parameters (Supplementary Figure 6). The first two axes of RDA explained 97.8 and 91.8% of the total variation in the dry and wet season, respectively. The environmental parameters that contributed both significantly to the bacterioplankton community–environment relationship were NO_3_^–^-N (*p* = 0.019/0.032) and Cl^–^ (*p* = 0.014/0.036) in the dry and wet season, respectively. The most abundant phylum *Proteobacteria* was significantly positively correlated with NO_3_^–^-N, while *Actinobacteria* was negatively (Supplementary Figure 5). *Bacteroidetes* was significantly negatively correlated with Cl^–^.

**FIGURE 3:**
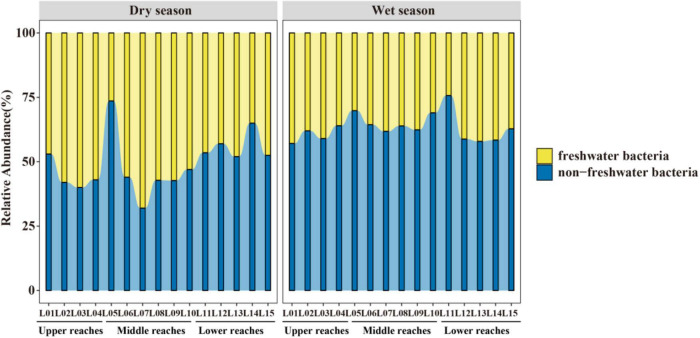
Relative bacterioplankton communities abundances of during the wet and dry seasons. Compositions are shown as freshwater and non-freshwater bacterial OTU distributions.

A considerable proportion of non-freshwater bacteria was commonly identified in both the dry and wet seasons (Figure 3). The relative abundances of non-freshwater bacteria during the wet season (62.94%) was significantly higher than during the dry season (49.33%) (*p* = 0.001). The proportion of non-freshwater bacteria was the highest in the region downstream of the Dexing Copper Mine (Y05, 73.61%). The abundance of non-freshwater bacteria in the dry season increased from upstream to downstream, however, non-freshwater bacterial abundances had large variations among sampling sites and were highest during the wet season in the middle reach communities. The proportion of non-freshwater bacteria was also higher in the vicinity of the Dexing Copper Mine (Y05, 69.83%) and was the highest in Leping county (Y11, 75.71%) communities. High input of allochthonous bacteria in the Dexing Copper Mine downstream and Leping counties created the local environmental bacteria pool.

### Deterministic and Stochastic Processes Along the HAILS Gradient and Their Relative Importance

**FIGURE 4:**
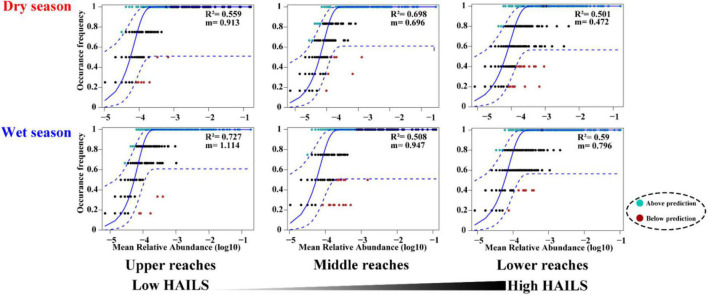
Neutral community model (NCM). The occurrence frequencies predicted for the dry season and wet season are displayed, which represents bacterioplankton communities for low, moderate, and large HAILS values in the Le’an River. The best fit to the neutral community model is given as solid blue lines (Chen et al., 2019), and the dashed blue lines indicate 95% confidence intervals. OTUs that occur more or less frequently than predicted are displayed in cyan and red, respectively. The *R*^2^ values indicate the model fit and *m* describes the immigration rate.

**FIGURE 5:**
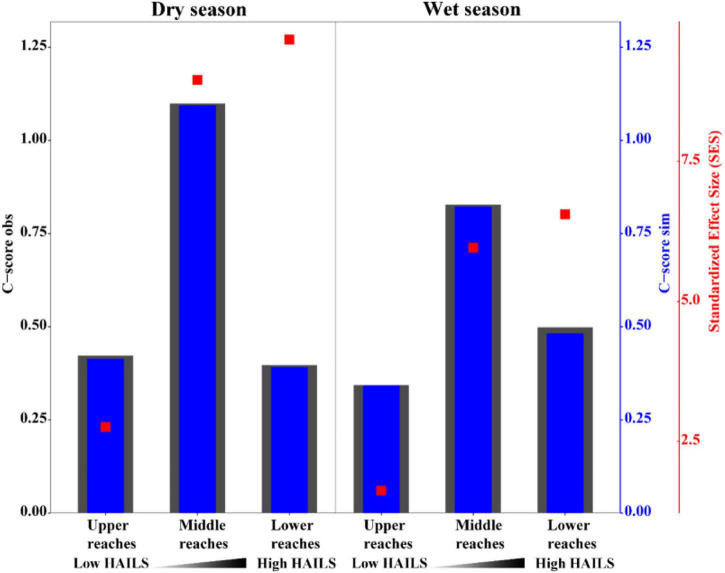
C-scores of null models. If observed C-scores are greater than simulated C-scores (i.e., C-score_*obs*_ > C-score_*sim*_) non-random co-occurrence is indicated. Standardized effect size less than –2 and greater than +2 represent aggregation and segregation, respectively.

**FIGURE 6:**
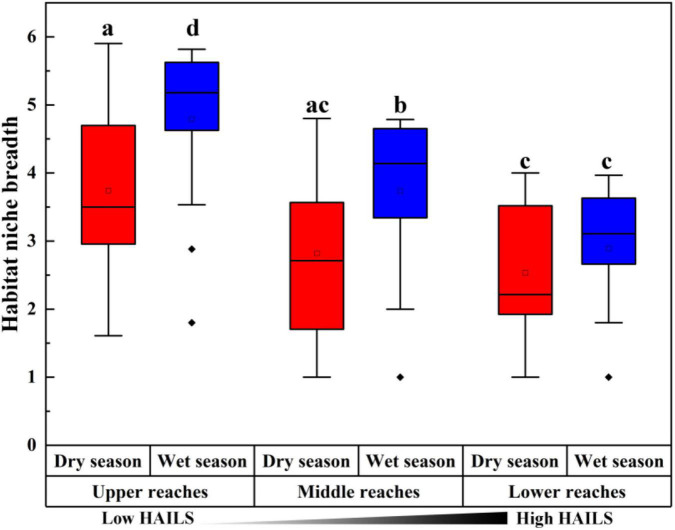
Mean habitat niche breadth comparison for all taxa along the HAILS gradient [different letters indicate significant differences at the *p* < 0.05 level (Mo et al., 2021)].

The fraction of the relationship between the occurrence frequency of OTUs and their relative abundance variations was estimated by a neutral community model (NCM; Figure 4). Stochastic processes showed increasing HAILS values (Figure 4), explaining 55.9%/72.7%, 69.8%/50.8%, and 50.1%/59% of the community variance during the dry and wet seasons, respectively. The m-value was higher for bacterioplankton taxa and decreased in the following order: upper reaches (*m* = 0.913/1.114)> middle reaches (*m* = 0.696/0.947)> lower reaches (*m* = 0.472/0.796) during the wet and dry season, respectively. Thus, stochastic processes are more important in shaping the bacterioplankton community assembly in the upper reaches. The C-scores revealed that the standardized effect size (SES) increased with increasing HAILS in both the dry and wet seasons, which suggests the greater importance of deterministic processes for bacterioplankton assemblage (Figure 5). In addition, all bacterioplankton communities (especially in the wet season) exhibited significantly wider niche breadths at low HAILS than at middle or high HAILS levels (Figure 6).

### Networks and Bacterioplankton Community Stability Along the HAILS Gradient

**FIGURE 7:**
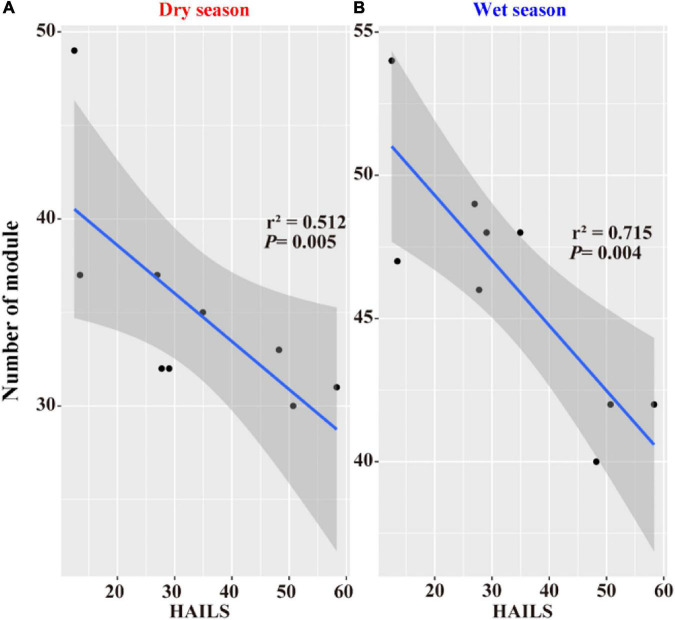
The stability-decay curves for the nine bacterioplankton molecular ecological networks in the Le’an River. Linear regression relationships are shown between the Number of module and HAILS in the **(A)** dry season and **(B)** wet season, respectively. The slopes of all lines were significantly less than zero and significantly different in pairwise comparison.

Distinct co-occurrence pattern networks were constructed based on all datasets from the Le’an River (Supplementary Figure 6). Nine networks along the HAILS gradient were analyzed to analyze how community structures changed with dynamic balancing of niche and neutrality during both seasons. These network data suggest that the majority of the bacterial networks in the wet season were more connected, larger, more modular, and were more often negatively correlated than in the dry season (Table 1). The topological properties of the networks also varied significantly with HAILS (Table 1). The average number of nodes (i.e., OTUs) in the lower reaches were 53.8%/40.9% higher than the upper reaches during the dry and wet season, respectively. Similarly, the links of the bacterial networks increased with increasing HAILS (i.e., network size increased with increasing intensity of human activity) in both the dry and wet seasons. This indicated that OTU associations are more frequently under environmental pressure than habitats distant from anthropogenic activities, which is also supported by the higher average clustering coefficients at high HAILS level (especially in the wet season). Additionally, networks in the upstream river have high modularity scores (49/54) in each season and the ratio of modularity in bacterial networks decreases significantly along the HAILS gradient (low, middle and high), indicating that the bacterial network is more robust and stable in the upper reaches (especially during the wet season) (Figure 7). Taxa were organized *via* loose connections which form hierarchical communities in suitable habitats, however, higher HAILS levels may disrupt this order, forcing communities to interact more among OTUs and merge small modules into larger ones.

**TABLE 1:** Parameters of bacterioplankton community network topology during the dry/wet season in the Le’an River.

Network indexes	Dry/Wet season								
	Upper reaches		Middle reaches				Lower reaches		
Total nodes (TN)	100/173	131/159	98/191	112/212	123/223	146/230	125/219	173/216	235/268
Total links (TL)	184/594	276/543	146/642	191/768	180/655	288/673	308/788	467/486	663/786
Negative links (NL)	49/267	135/216	50/288	76/351	54/279	114/269	107/283	110/204	189/380
Positive links (PL)	135/327	141/327	96/354	115/417	126/376	174/404	201/505	357/282	474/406
Negative/Positive (NP)	0.36/0.82	0.96/0.66	0.52/0.81	0.66/0.84	0.43/0.74/	0.66/0.67	0.53/0.56	0.31/0.72	0.40/0.94
R square of power-law (R)	0.51/0.15	0.19/0.08	0.54/0.10	0.61/0.09	0.92/0.32	0.53/0.36	0.30/0.23	0.47/0.36	0.34/0.24
Average degree (avgK)	3.68/6.87	4.21/6.83	2.98/6.72	3.41/7.25	2.93/5.87	3.95/5.85	4.93/7.20	5.40/4.50	5.64/5.87
Average clustering coefficient (avgCC)	0.59/0.64	0.69/0.65	0.59/0.70	0.54/0.71	0.58/0.75	0.71/0.70	0.66/0.68	0.62/0.66	0.71/0.80
Average path distance (APD)	1.05/1.26	1.25/1.06	1.02/1.21	1.35/1.07	1.37/1.12	1.08/1.21	1.17/1.27	2.24/1.28	1.45/1.16
Centralization of degree (CD)	0.07/0.10	0.07/0.09	0.07/0.06	0.07/0.08	0.04/0.06	0.05/0.05	0.10/0.08	0.10/0.04	0.06/0.04
Graph density (GD)	0.04/0.04	0.03/0.04	0.03/0.04	0.03/0.03	0.02/0.03	0.03/0.03	0.04/0.03	0.03/0.02	0.02/0.02
Number of module (NM)	49/54	37/47	32/46	37/49	35/48	32/48	33/40	31/42	30/42
Modularity (M)	0.85/0.90	0.71/0.92	0.73/0.79	0.89/0.87	0.91/0.87	0.86/0.80	0.82/0.82	0.84/0.71	0.82/0.70

Summarizing all networks, the nodes with the top three highest degrees were *Proteobacteria, Bacteroidetes*, and *Actinobacteria*, being potential keystone species (Supplementary Figure 6). Hubs and connectors are defined here as keystone species, meaning that if these taxa were removed, the associated modules and networks may also dissipate. No node in the bacterial network in the upper, middle, and lower reaches falls in under the classification of network hubs or connectors (Supplementary Figure 7) for either season. Nearly all of the nodes are peripheral modules except for one network in the middle in which is a module hub (*Proteobacteria*). This indicated that *Proteobacteria, Bacteroidetes*, and *Actinobacteria* were the dominant phyla in bacterial networks and were unaltered spatially and temporally.

### Environmental Factors Related to Bacterioplankton Community and Network Variation

Geographic distance can also influence the structure of bacterioplankton assembly and were thus evaluated (Supplementary Figure 8). Communities showed a strong distance-decay pattern, in which community dissimilarity increased with distance, suggesting that bacterioplankton taxa under different hydrologic regimes (seasons) exhibited similar spatial patterns. Community differences were also well described by river length (*r* = 0.476, *p* = 0.001 in the dry season; *r* = 0.603, *p* = 0.001 in the wet season). The bacterioplankton communities exhibited a stronger distance-decay pattern in the wet season than in the dry season.

Determining the relationship between environmental factors and community structures is critical. Bioenv analyses (Table 2) indicted that the optimal subset of land use types for the bacterioplankton composition are forests (Bioenv correlation = 0.3369 in the dry season; Bioenv correlation = 0.4738 in the wet season). The optimal subset of land use types for the bacterioplankton networks are forests and freshwater regions (Bioenv correlation = 0.6157) in the dry season. Farmland, forest, and urban levels were optimal for bacterioplankton composition (Bioenv correlation = 0.4906) in the wet season (Table 3). Optimal water chemistry parameters are NO_3_^–^-N and Cl^–^ (Bioenv correlation = 0.6824) in the dry season. Cl^–^ concentrations (Bioenv correlation = 0.6620) were optimal to bacterioplankton network in the dry season. NO_3_^–^-N levels were optimal for bacterioplankton composition (Bioenv correlation = 0.6719) in the wet season, Cl^–^, Cd, NO_3_^–^N and TOC (Bioenv correlation = 0.5099) were optimal for bacterioplankton networks in the wet season. River length and mean dendritic stream length were the optimal geographic distance parameters for both bacterioplankton composition and network.

**TABLE 2:** Correlations of different combinations of environmental factors and bacterioplankton communities, as determined by Bioenv analysis.

	Combination in dry season	Correlation	Combination in wet season	Correlation
Land use	Forest	**0.3369**	Forest	**0.4738**
	Farmland + Forest	0.2391	Forest + Freshwater	0.4336
	Farmland + Forest + Freshwater	0.1932	Farmland + Forest + Freshwater	0.4304
	Farmland + Forest + Freshwater + Urban	0.1770	Farmland + Forest + Freshwater + Urban	0.3752
Water chemistry	NO_3_^–^-N	0.6312	NO_3_^–^-N	**0.6719**
	NO_3_^–^-N + Cl^–^	**0.6824**	NO_3_^–^-N + Cl^–^	0.6248
	NO_3_^–^-N + Cl^–^ + As	0.6268	NO_3_^–^-N + Cl^–^+ FNU	0.5938
	NO_3_^–^-N + Cl^–^ + As + Cd	0.6343	NO_3_^–^-N + Cl^–^ + FNU + NH_4_^+^-N	0.5841
Geographic distance	River length	**0.4764**	River length	0.6027
	River length + Mean dendritic stream length	0.4716	River length+ Mean dendritic stream length	**0.6082**
	River length + Mean dendritic stream length + Cumulative dendritic distance	0.4461	River length+ Mean dendritic stream length + Cumulative dendritic distance	0.5799
	River length + Mean dendritic stream length + Cumulative dendritic distance + Catchment area	0.4138	River length + Mean dendritic stream length + Cumulative dendritic distance + Catchment area	0.5581

*Maximum values are indicated by bold text.*

**TABLE 3:** Correlations of different combinations of environmental factors and bacterioplankton network indexes (TN, TL, avgK, avgCC, GD, NM, and M), as determined by Bioenv analysis.

	Combination in dry season	Correlation	Combination in wet season	Correlation
Land use	Forest	0.5761	Forest+ Grassland	0.4677
	Forest + Freshwater	**0.6157**	Farmland + Forest + Urban	**0.4906**
	Forest + Freshwater + Other	0.6021	Farmland + Forest + Grassland + Urban	0.4824
	Forest + Freshwater + Urban+ Other	0.5997	Farmland + Forest + Grassland + Urban + Freshwaters	0.4569
Water chemistry	Cl^–^	**0.6620**	Cl^–^ + Cd	0.5097
	Cl^–^ + Cd	0.4561	Cl^–^ + Cd + TP	0.5022
	Cl^–^ + Cr +TOC	0.3194	Cl^–^ + Cd + NO_3_^–^-N + TOC	**0.5099**
	Cl^–^ + Cr +TOC + Co	0.2463	Cl^–^ + Cd + NO_3_^–^-N + TOC +pH	0.5042
Geographic distance	River length	**0.4860**	Mean dendritic stream length	**0.5122**
	River length + Cumulative dendritic distance	0.4404	River length + Mean dendritic stream length	0.5079
	River length + Cumulative dendritic distance + Catchment area	0.4353	River length + Mean dendritic stream length + Catchment area	0.4757
	River length + Cumulative dendritic distance + Catchment area+ Mean dendritic stream length	0.4154	River length + Mean dendritic stream length + Catchment area + Cumulative dendritic distance	0.4662

*Maximum values are indicated by bold text.*

**FIGURE 8:**
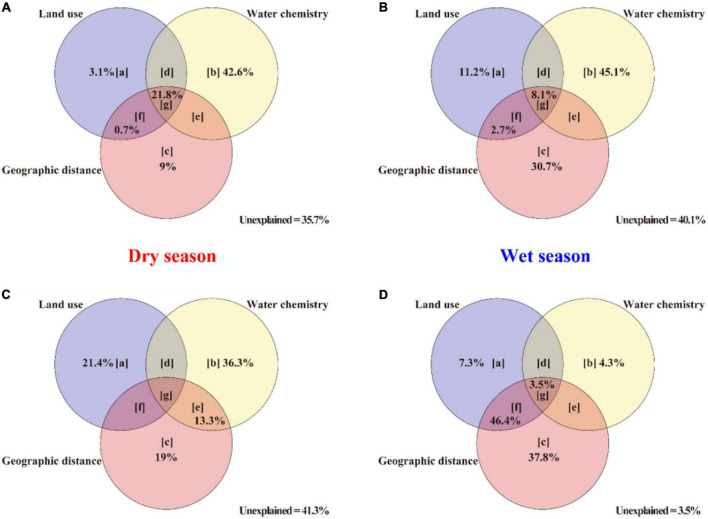
Variation partitioning for Le’an River bacterioplankton communities. The effects on **(A)** dry season composition, **(B)** wet season composition, **(C)** dry season networks, and **(D)** wet season networks are evaluated based on land use, water chemistry, and geographic distance parameter contributions (see Supplementary Figure 6). Panels [a], [b], and [c] represent pure contributions of individual explanatory matrices; panels [d], [f], and [e] represent the combined contributions of two explanatory matrices; panel [g] represents the joint contribution of three explanatory matrices. “Unexplained” indicates the proportion of the variation which was not explained by any of our parameters.

Variance partitioning (Figure 8) suggests that the bacterioplankton composition and network are better explained by geographic distance and land use than water chemistry during the wet season. However, more environmental factors are found to regulate the composition and stability of the interaction network of the planktonic bacterial community in the Le’an River in the dry season, which shows that water chemistry had more influence on the bacterioplankton composition and networks than geographic distance and land use in this instance (Supplementary Figure 9). The variation in community composition that was explained by water chemistry patterns, 42.6% in bacterioplankton composition in the dry season and 45.1% in the wet season and 36.3% in bacterioplankton network in the dry season, was higher than the impacts from other environmental factors (Figure 8). However, the effects of land use patterns and geographic distance on community variations, 44.6% in bacterioplankton composition and 91.5% in bacterioplankton network, is higher than that of water chemistry (30.2 and 90.3%) in the wet season.

## Discussion

The richness and diversity of bacterioplankton slightly increases with increasing anthropogenic stresses along the human activity intensity spatial gradient. Our results support previous observations that the richness and diversity of bacterioplankton is higher under human influenced urban conditions (Altermatt et al., 2013; Huang and Huang, 2019; Wang et al., 2019). River discharge variations in different hydrological seasons can yield significant changes in water chemistry and chemical pollutants as well resulting from allochthonous inputs (e.g., sediment resuspension and erosion) (Hu et al., 2017). We found that bacterioplankton communities exhibited distinct distribution patterns across space rather than by seasonality, likely due to clear increases in anthropogenic pressure present in the downstream regions but absent in the upstream regions (Supplementary Figure 4). The middle and lower reaches of the Le’an River have experienced rapid urbanization, characterized by increased building and farmland land use (Figure 1 and Supplementary Table 1). This is especially true in sites with abundant urban areas (e.g., Dexing Copper Mine Y05 and Leping city Y11), as non-freshwater bacteria were abundant in the water communities (Figure 3). Such non-freshwater populations can be introduced through runoff and anthropogenic input (Staley et al., 2013). Newly introduced allochthonous bacteria can be capable of proliferating in their new lotic environment, at least temporarily, allowing them to be constitutive community members (Savio et al., 2015). Furthermore, the agricultural and urban watersheds are more influenced by increased human activity which can result in higher nitrogen and phosphorus concentrations. This in turn can support a greater variety of bacterioplankton, stimulate autochthonous bacterial growth and enhance community richness (Mohit et al., 2014; Roberto et al., 2018). Bacteria derived from sewage can also contribute to bacterial community abundance and diversity due to their higher growth rates (Shu et al., 2020). Additionally, allochthonous bacterial input, evolution, and spreading along the river may be strengthened by various human activities (i.e., agricultural land use may be more intensive in downstream areas, and towns or mines may influence middle reaches). However, inconsistent results were reported in the Shaying River basin (Li et al., 2020), where increased human land use reduces bacterioplankton biodiversity and ecosystem functions. This disagreement may be due to the sampling of different waterbodies under multiple disturbances.

Community composition may be due to environmental condition variations influenced by land use at the local or regional level. Figure 2 supports the findings of other studies conducted in other subtropical rivers, where a dominant role of *Proteobacteria* were also observed (Ouyang et al., 2020; Wang Y. et al., 2020). The richness and abundance of *Proteobacteria* significantly rose along the HAILS gradient and with increasing anthropogenic stress in the dry season (Figure 2). This may be attributed to variations in environmental conditions influenced by land use. Anthropogenic activity along rivers may increase the quantities of nutrients and change the form and proportion of nutrients and physicochemical variables (Yang et al., 2022). The concentrations of NO_3_^–^-N, NH_4_^+^-N and TOC were higher in the dry season than in the wet season, and NO_3_^–^-N, NH_4_^+^-N and TP in the downstream regions were significantly higher than in the upstream regions due to the influence of mines and agricultural activities (Supplementary Figure 1). The higher nitrogen content may lead to increases in the relative abundances of *Proteobacteria* (Burkert et al., 2003). The highest bacterioplankton community abundance in the wet season was *Actinobacteria*, which is correlated with less eutrophic conditions (Haukka et al., 2006). Increased precipitation may dilute the river nutrient concentration (e.g., NO_3_^–^-N) and induce an increase in the relative abundances of *Actinobacteria* (Supplementary Figure 5). Conversely, *Cyanobacteria* were the third most abundant phylum during the wet season but had very low abundance in the dry season. *Cyanobacteria* have a total phosphorus (TP) threshold of 0.010 mg/L and the probability that *Cyanobacteria* will become dominant over other bacterioplankton species increases with increasing TP to a maximum probability of about 80% when water TP reaches or exceeds 0.100 mg/L (Bridgeman et al., 2012). The reason for *cyanobacteria* being the third most abundant phylum during the wet season is likely attributed to the high level of TP. *Bacteroidetes* are often enriched in the intestines of humans or animals and can be effective fecal indicators (Ibekwe et al., 2016; Shu et al., 2020). Thus, *Bacteroidetes* are detected consistently across all the sites in both the dry and wet seasons, suggesting that they came from anthropogenic inputs (Dubinsky et al., 2012). These microbes can be active in microbe-nutrient interactions.

Human activity intensity in freshwater environments can significantly influence bacterioplankton community assembly, through impacts on deterministic and stochastic process balance. Stochastic and deterministic processes work concurrently and symbiotically in natural ecosystems. We found here that stochastic processes are more important in shaping the bacterioplankton community assembly in the upper reaches and the species dispersal of bacterioplankton taxa was higher in the wet season than in dry seasons (Figure 4). When the stable environments in the upper reaches are disturbed by anthropogenic activity, niche-based selection strengthens and bacterioplankton communities become less characterized by stochasticity. Freshwater bacterioplankton can become exposed to lower physiological stress at low human activity intensity levels, and they can then grow and reproduce more freely, which results in the dominance of stochastic processes (Wu B. et al., 2021). Jiao S. et al. (2020) found that in low environmental stress ecosystems which experience lower environmental heterogeneity or under less competitive interactions between environmental generalists, stochastic assembly mechanisms can overrule deterministic processes. Conversely, strong selective pressure may be experienced by freshwater bacterioplankton when human activity intensity increases. Dini-Andreote et al. (2015) found that increased allochthony may lead to an increase in stochastic processes during the wet season under other environmental factors which do not impose strong selection. Our C-score results demonstrated that the value of standardized effect size (SES) increased with anthropogenic intensity, suggesting that community assembly is more strongly by deterministic processes under such activity (Figure 5). When environmental pressure increases dramatically, due to such things as alkalization, acidification, or eutrophication, important taxa activities that are sensitive to these pressures (especially immigrants with small populations) are suppressed (Wang L. et al., 2021). Thus, neutrality is weakened, and niche-based selection leads the community as human activity intensity increases. In addition, the bacterioplankton communities show wider niche breadths (especially in the wet season) under low human activity intensity than at the medium/high human activity intensity conditions (Figure 6). This indicates that community assembly is more influenced by deterministic processes at high human activity intensity. This may be since deterministic processes tend to have a stronger effect on habitat specialists with a narrow niche breadth than on generalists with a wide niche breadth (Pandit et al., 2009).

Human activity intensity promotes destabilizing properties in the bacterioplankton interactive network. Network modules may act as network stability indicators of important ecological processes following the disturbance of human activity intensity changes (Yuan et al., 2021). Therefore, our results demonstrate that an increase in human activity intensity of about 10%/10% will reduce co-occurrence network stability of bacterioplankton community by an average of 0.62%/0.42% in the dry and wet seasons, respectively (Figure 7). The greater the number of modules, the more bacterioplankton taxa distributed in multiple tiny modules in the upper reaches. This indicates that multiple inter-OTU communications may have been obstructed (Feng et al., 2014). Accordingly, an individual disturbance might be constrained to only one small module, which minimizes other modules, and thus the stability of bacterioplankton co-occurrence networks can improve (Liu Z. et al., 2018). Conversely, for the assemblages living through heavy influence of the mining (e.g., the Dexing Copper Mine) areas or farmlands, the networks were discerned with high average clustering coefficients, average degrees, and less numbers of module (Table 1). A community network under strong anthropogenic activity would break down the boundaries between different modules, strengthen the interconnections between different bacterioplankton taxa, and enhance resource transfer efficiency, so that they would be able to cope with environmental perturbations through mutual cooperation (Deng et al., 2012). This result is similar to that reported by Wu M. et al. (2021). The interactions among bacterioplankton taxa can intensify (higher connectivity, more nodes, and links), increasing the connectivity among modules, and merging them into fewer but larger modules which ultimately decrease the modularity and stability of the network.

The ratio of modularity in the bacterial networks decreased significantly along human activity intensity spatial gradient, consistent with the general fitness tendency of NCM. The more modules, the more niches overlap between each other, and a higher overlap of niches produces neutral scenario (Banerjee et al., 2016; Carmel et al., 2017). Consequently, networks of more neutrally assembled communities (especially in the upper reaches) likely have a greater number of modules than those mainly shaped by deterministic selection and maintain a stable structure for neutrally assembled communities in low interference condition. In addition, our results indicate that bacterioplankton communities in the upper reaches had wider niche breadths than the middle and lower reaches (Figure 6), meaning that they can adapt to many environmental niches (Mo et al., 2021). This pattern is closely associated with bacterioplankton diversity and richness. Thus, human activity intensity may affect bacterioplankton diversity and richness, and consequently impacts network structure.

Select few keystone taxa play pivotal roles in the construction of bacterioplankton communities. Module hubs and connectors may be considered keystone species, as they play important roles in maintaining network structure relative (Tylianakis and Morris, 2017). The disappearance of these keystone taxa may cause modules and networks to disassemble, thus keystone taxa likely support ecosystem stability (Wang X. et al., 2021). The large majority of nodes are classified as peripheral modules except for one network in the middle reaches in which is classified as a module hub (*Proteobacteria*). The nodes with the top three highest degrees indicated that *Proteobacteria, Bacteroidetes*, and *Actinobacteria* were the dominant phyla and keystones in the Le’an River bacterial networks and were unaltered by spatial and temporal distribution (Supplementary Figure 6). Dominant phyla can impact ecosystem exclusively by virtue of sheer abundance (Banerjee et al., 2018). Keystones and module hubs can play the same roles in the dynamics of communities; in particular, keystones are crucial in maintaining taxa coexistence and adapting communities to their environments.

Water chemistry had a stronger influence on bacterioplankton communities (especially composition) than did geographic distance and land use in the dry season in the Le’an River. Likely sources of NO_3_^–^-N include wastewater from mining, agricultural, and domestic sources (Wang et al., 2019). Cl^–^ is also a common indicator of domestic wastewater (Lim et al., 2017). Strong influences of collective allochthonous input is also suggested by significant positive correlations between NO_3_^–^-N concentration and other water chemistry parameters (Supplementary Figure 9). Half of our water chemistry parameter levels were significantly larger in the dry season than in the wet season (Supplementary Table 2 and Supplementary Figure 1). Nutrient concentrations can have a considerable influence on bacterioplankton composition since nutrient concentration changes directly impact substrate concentrations and bacterial nutrient metabolism (Sun et al., 2021). Thus, the dominance of environmental factors in bacterioplankton composition variations may be attributed to decreasing water levels in the dry season, which results in higher nutrient (e.g., nitrogen) concentrations.

The bacterioplankton community (especially the bacterioplankton networks) is more influenced by geographic distance and land use than the water chemistry in the wet season. Geographic distance and land use contributes to community variation more than other environmental factors (Figure 8). Forest areas are the most important and best subset of land use variables to explain bacterioplankton community (especially the bacterioplankton composition) variations (Tables 2, 3). The reduction of forest land in the middle and lower reaches can increase soil erosion, sediment transport and the abundance of non-freshwater bacteria attached to the river, and therefore intensify the interactions among bacterioplankton taxa (Bussi et al., 2017). In the wet season, river length and mean dendritic stream length are the best geographic distance parameter subsets to explain bacterioplankton community dissimilarities (Table 2). The influence of river flow can also be significant. Water levels are higher in the wet season, and as river flow increases, the river connectivity and habitat homogeneity can also increase, which can assist the passive movement of microorganisms over great distances (Wang et al., 2015). Smaller bacterioplankton taxa disperse more easily and cannot readily counteract downstream flow. A more significant distance-decay relationship can also support the significant effect of geographic distance in the wet season (Supplementary Figure 7). Liu T. et al. (2018) found that mean dendritic stream length best explained the spatial similarity of bacterioplankton communities in the Yangtze River. Domestic wastewater is an important source of allochthonous bacteria, confirmed by the importance of NO_3_^–^-N and Cl^–^ in community variation (Table 2). In the wet season, the input of allochthonous bacteria increases due to runoff from multiple sources (Wang et al., 2019). Thus, the influence of geographic distance and land use also increases in the wet season. In addition, much of the unexplained variation could be attributed to other biotic interactions, such as competition, trophic interactions, and unmeasured environmental or biological factors (He et al., 2021).

## Conclusion

Deterministic and stochastic processes can both be important in bacterioplankton communities. Here, we merge these conceptual mechanisms along with co-occurrence networks into bioinformatic analyses of bacterioplankton communities and find that deterministic processes can increase species richness and diversity. This can be particularly true along the spatial gradient of human activity intensity. However, the influence of stochastic processes diminishes, and niche-based selection imposes more constraints on communities. We also find the magnitude of stability in bacterioplankton community networks along the human activity intensity spatial gradient that was quantitatively associated with human activity. Water chemistry (especially NO_3_^–^-N and Cl^–^) is the major driver of bacterioplankton composition and networks during the dry season, while geographic distance (especially river length and mean dendritic stream length), and land use (especially forest regions) are the major drivers in the wet season. However, further investigations are still required to better elucidate these processes and the reliability of the factors determined here, such as consideration of seasonal and yearly assessment of bacterioplankton taxa.

## Data Availability Statement

Raw reads were deposited in the NCBI Sequence Read Archive database, access number: SRP142494.

## Author Contributions

PW designed the study. PW, BW, AD, YS, JZ, YX, YH, LC, HZ, MN, and MD collected the samples. BW analyzed the genetic data. BW, AD, YS, JZ, YX, YH, LC, HZ, MN, and MD analyzed the geographic data. PW and BW wrote the manuscript with contributions from all co-authors. All authors have approved the final manuscript for publication.

## Conflict of Interest

The authors declare that the research was conducted in the absence of any commercial or financial relationships that could be construed as a potential conflict of interest.

## Publisher’s Note

All claims expressed in this article are solely those of the authors and do not necessarily represent those of their affiliated organizations, or those of the publisher, the editors and the reviewers. Any product that may be evaluated in this article, or claim that may be made by its manufacturer, is not guaranteed or endorsed by the publisher.

## Footnotes

1
http://www.geodata.cn

2
http://sourceforge.net/projects/rdp-classifier/

3
http://psbweb05.psb.ugent.be/conet
