# Supplementary material for: Anthropogenic Intensity-Determined Assembly and Network Stability of Bacterioplankton Communities in the Le’an River
Source: Front Microbiol. 2022 May 4;13:806036. doi: 10.3389/fmicb.2022.806036 (PMC9114710; doi:10.3389/fmicb.2022.806036)
Supplement: Supplementary file 1 [file Data_Sheet_1.docx]

# Supplementary information

**Table S1**

Location information and HAILS level (see Equ (1)) of land use patterns for the 15 sampling sites considered in the Le’an River basin.

| Region | Water sampling site | Farmland  area percentage（%） | Forest  area percentage（%） | Grassland  area percentage（%） | Freshwaters  area percentage（%） | Urban  area percentage（%） | Other  area percentage（%） | HAILS  （%） |
| --- | --- | --- | --- | --- | --- | --- | --- | --- |
| Upper reaches | L01 | 10.20 | 87.83 | 0.47 | 0.90 | 0.60 | 0.00 | 12.80  (1.38)^c^ |
|  | L02 | 11.24 | 85.36 | 0.43 | 0.57 | 2.41 | 0.00 |  |
|  | L03 | 12.06 | 85.66 | 0.51 | 0.85 | 0.92 | 0.00 |  |
|  | L04 | 12.02 | 79.92 | 0.55 | 5.76 | 1.76 | 0.00 |  |
| Middle reaches | L05 | 16.21 | 78.32 | 0.98 | 0.78 | 3.71 | 0.00 | 28.39  (12.43)^b^ |
|  | L06 | 18.35 | 67.65 | 1.94 | 5.61 | 6.44 | 0.00 |  |
|  | L07 | 31.76 | 54.03 | 5.21 | 2.26 | 6.74 | 0.00 |  |
|  | L08 | 14.19 | 79.65 | 1.89 | 0.87 | 3.40 | 0.00 |  |
|  | L09 | 40.27 | 41.11 | 6.53 | 3.66 | 8.43 | 0.00 |  |
|  | L10 | 19.98 | 74.86 | 2.58 | 1.72 | 0.86 | 0.00 |  |
| Lower reaches | L11 | 29.74 | 61.06 | 4.78 | 2.15 | 2.21 | 0.07 | 52.09  (18.38)^a^ |
|  | L12 | 72.34 | 4.38 | 8.69 | 10.51 | 4.07 | 0.00 |  |
|  | L13 | 34.62 | 46.02 | 8.36 | 8.39 | 1.69 | 0.93 |  |
|  | L14 | 56.17 | 20.74 | 10.01 | 7.03 | 6.05 | 0.00 |  |
|  | L15 | 50.16 | 31.32 | 9.74 | 5.35 | 3.44 | 0.00 |  |

* HAILS is the mean of four, five, or six sampling sites, with standard errors given in parentheses. Superscript letters indicate statistical significance at (*p* < 0.05). One‐way ANOVA tests were performed in three regions (n=3).

**Table S2**

Hydrochemical parameters of the Le'an River.

|  | Season | |  | Region | | |
| --- | --- | --- | --- | --- | --- | --- |
|  | Dry season  n=15 | Wet season  n=15 |  | Upper reaches n=8 | Middle reaches n=12 | Lower reaches n=10 |
| pH | 7.33±0.65 | 7.19±0.16 |  | 7.74±0.48^a^ | 7.11±0.36^b^ | 7.05±0.28^b^ |
| EC (μs cm-^1^) | 259.93±138.61 | 191.07±90.00 |  | 80.75±25.94 ^a^ | 281.00±116.74^bc^ | 274.70±61.95^ac^ |
| NO_3_^-^-N (mg L^-1^) | 1.30±0.10^a^ | 0.51±0.23^b^ |  | 0.53±0.29^c^ | 1.06±1.18^c^ | 1.02±0.29^a^ |
| NH_4_^+^-N (mg L^-1^) | 0.70±0.87^a^ | 0.20±0.14^b^ |  | 0.18±0.14^c^ | 0.51±0.92^c^ | 0.59±0.53^a^ |
| TOC (mg L^-1^) | 7.27±1.45 | 6.03±2.01 |  | 5.62±1.83 | 7.08±1.79 | 6.96±1.74 |
| TP (mg L^-1^) | 0.08±0.08 | 0.09±0.10 |  | 0.05±0.03 | 0.08±0.10 | 0.11±0.11 |
| Cl^-^ (mg L^-1^) | 9.96±10.84 | 6.35±5.85 |  | 1.60±0.33^c^ | 5.34±3.72^b^ | 16.79±9.92^a^ |
| SO_4_^2-^ ( mg L^-1^) | 64.11±58.05 | 45.69±37.49 |  | 6.23±5.22^b^ | 88.06±56.57^a^ | 54.04±14.33^a^ |
| Cr (ug L^-1^) | 1.37±0.45^a^ | 0.99±0.39^b^ |  | 1.04±0.51 | 1.29±0.58 | 1.16±0.16 |
| Cu (ug L^-1^) | 5.11±2.16^a^ | 2.19±1.25^b^ |  | 2.36±1.88^b^ | 4.40±2.48^a^ | 3.77±2.06^a^ |
| Zn (ug L^-1^) | 25.21±12.62^a^ | 6.99±4.53^b^ |  | 11.91±10.00 | 19.50±15.78 | 15.38±11.93 |
| Cd (ug L^-1^) | 0.53±0.50^a^ | 0.07±0.06^b^ |  | 0.04±0.04^b^ | 0.36±0.37^a^ | 0.44±0.56^a^ |
| Fe (ug L^-1^) | 79.86±37.81 | 53.21±27.09 |  | 45.26±28.25 | 83.13±44.09 | 63.64±13.51 |
| Co (ug L^-1^) | 1.22±3.00^a^ | 0.07±0.06^b^ |  | 0.05±0.03 | 1.37±3.37 | 0.26±0.23 |
| As (ug L^-1^) | 2.07±0.85 | 2.17±1.75 |  | 1.00±0.43^b^ | 2.92±1.65^a^ | 2.04±0.66^a^ |
| Pb (ug L^-1^) | 1.71±3.47^a^ | 0.35±0.32^b^ |  | 2.25±4.80 | 0.70±0.68 | 0.45±0.30 |

Note: Each value is the mean of fifteen, eight, ten, or twelve sampling replicates, along with standard errors. Superscript letters indicate statistical significance at (*p* < 0.05). One‐way ANOVA tests were performed in two seasons (n = 15/15, respectively) and three regions (n = 8/12/10, respectively).

**
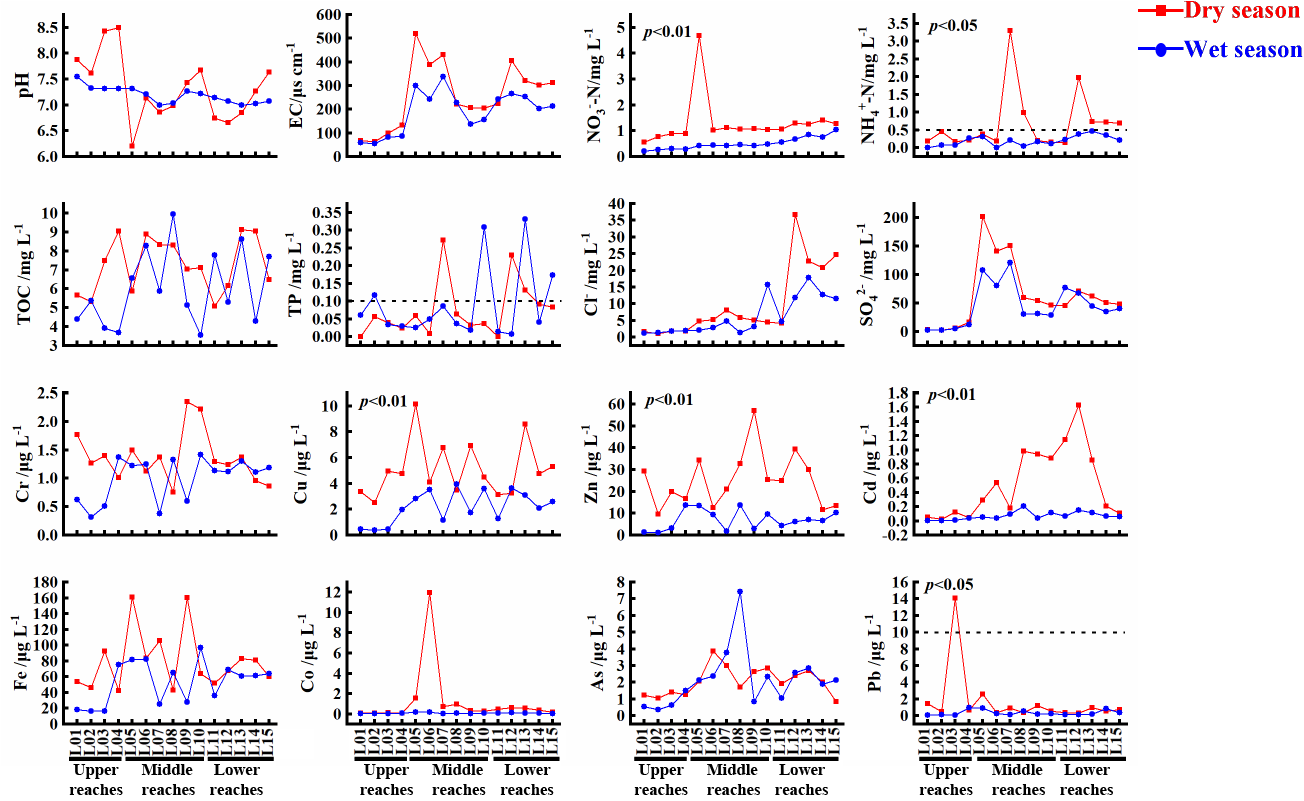
**

**FigureS1.** Water chemistry parameter measurements arranged by site from the upper reaches to the lower reaches.


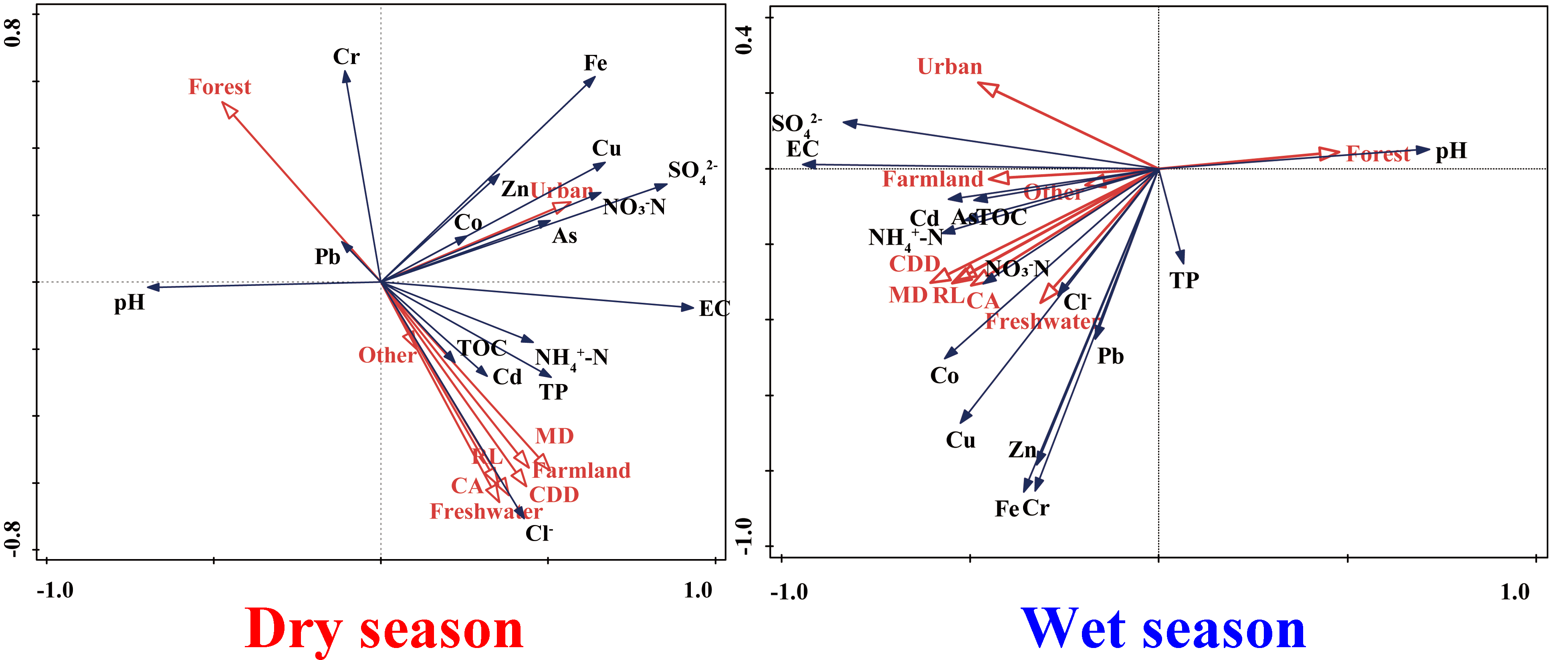


**FigureS2.** The RDA biplots showing the relationships between water chemistry variables, geographic distance parameters and land use in the dry and wet season.

**Table S3**

Number of OTUs and taxonomic groups for bacterioplanktonic communities in the Le’an River.

| Taxa | OTUs | Phylum | Class | Order | Family | Genus | Species |
| --- | --- | --- | --- | --- | --- | --- | --- |
|  | Richness |  |  |  |  |  |  |
| Dry season | 620  655  770 | 28 | 58 | 101 | 169 | 279 | 419 |
| Wet season |  | 26 | 55 | 99 | 172 | 274 | 411 |
| Whole |  | 30 | 64 | 110 | 188 | 310 | 478 |


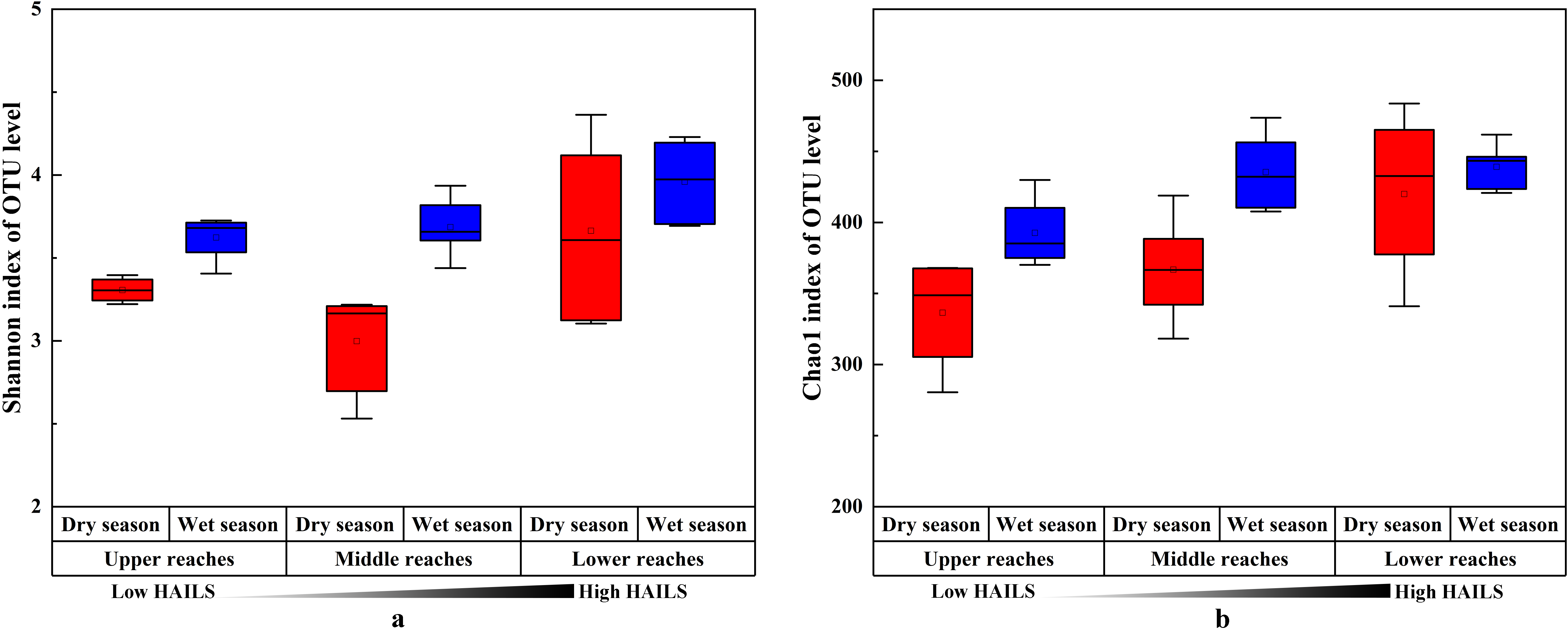


**FigureS3.** Comparison of Shannon diversity indices (a) and Chao1 richness (b) of communities for the upper and downstream reaches of the Le’an River. Boxes show mean values ± SE, while whiskers show means ± SD. Dry season distributions are indicated by red and wet season distributions by blue.





**FigureS4.** Spatio-temporal variations of bacterioplankton community structure in the Le’an River, China. Principal coordinate analyses (PCoA) with Bray-Cutris distance matrices of bacterioplankton communities were calculated for each of the three reaches (a) during the dry and wet seasons (b), respectively.


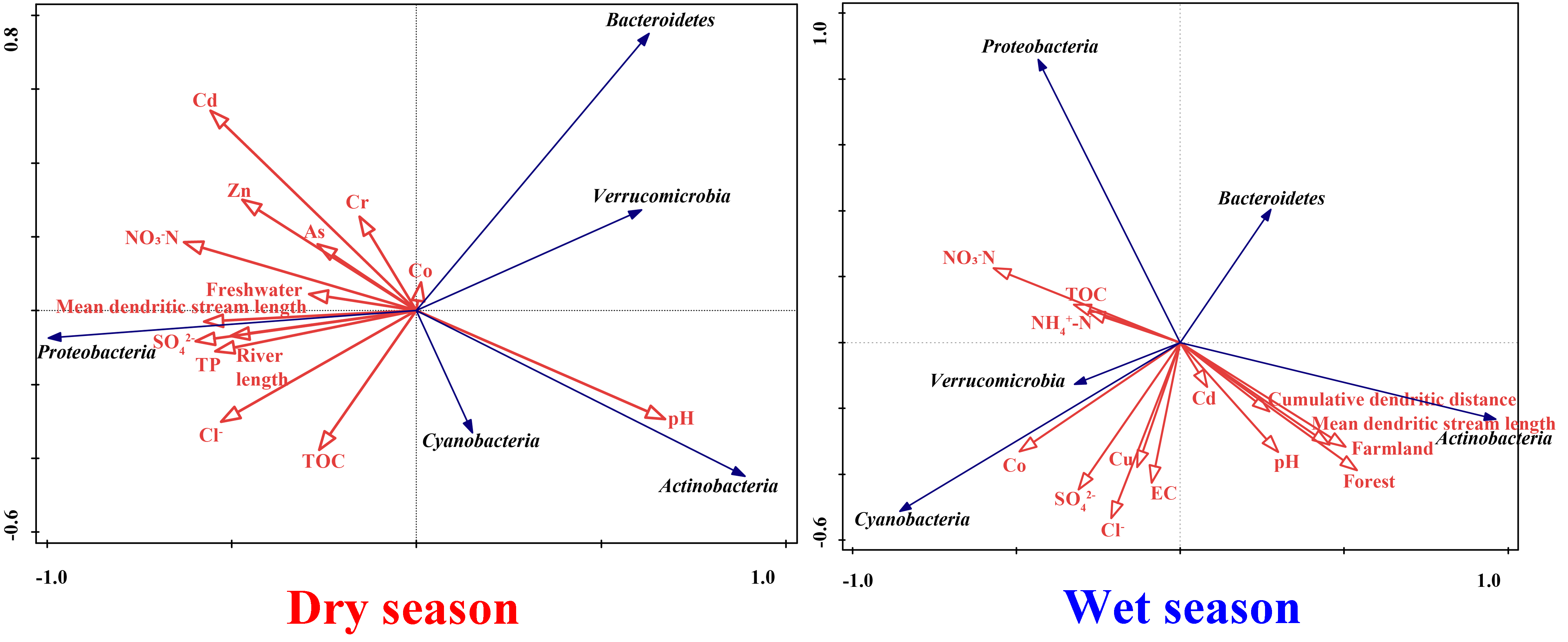


**FigureS5.** The RDA biplots showing the relationships between the bacterioplankton phyla and environmental parameters in the dry and wet season.


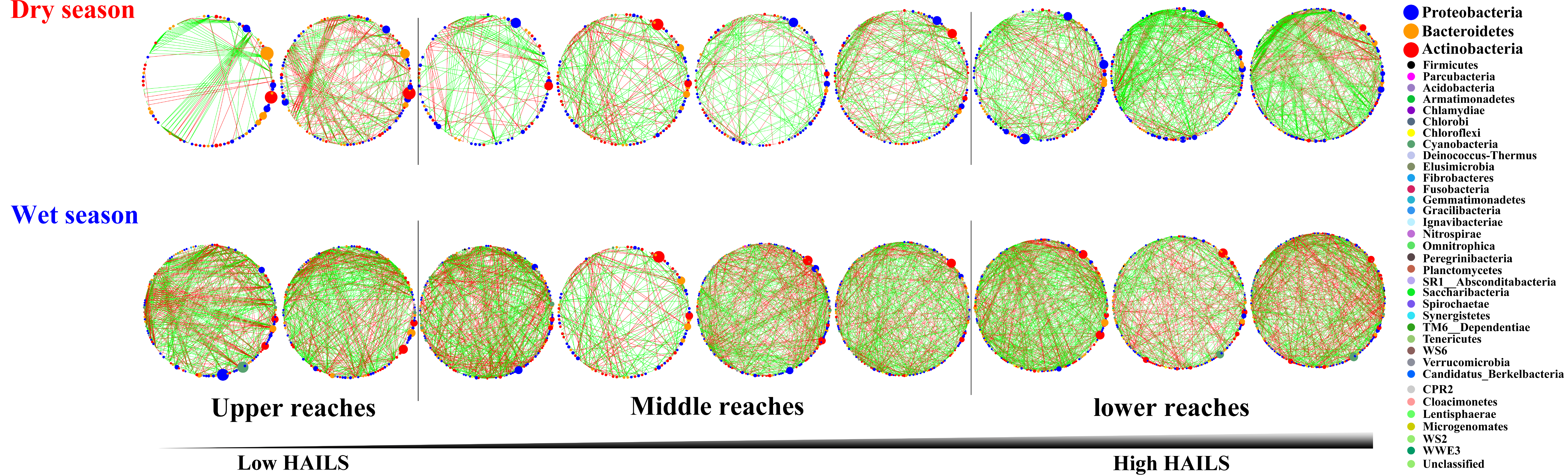


**FigureS6.** Bacterial community molecular ecological networks during the wet and dry season in the Le'an River. Significant positive (Spearman correlation, *p* <0.05, *r* >0.8) and negative (Spearman correlation, *p* <0.05, *r* <−0.8) linear relationships are indicated by green and red lines, respectively. Circle size represents the relative abundances of bacteria, and the black line represents categorical stress rankings.
