# Supplementary material for: Anthropogenic Intensity-Determined Assembly and Network Stability of Bacterioplankton Communities in the Le’an River
Source: Front Microbiol. 2022 May 4;13:806036. doi: 10.3389/fmicb.2022.806036 (PMC9114710; doi:10.3389/fmicb.2022.806036)
Supplement: Supplementary file 2 [file Data_Sheet_2.docx]

**FigureS7.** Topological roles of bacterioplankton during the wet and dry season in the Le'an River**.**

Note：Network hubs, module hubs, and connectors were termed keystone network topological features; these are considered to play important roles in the stability and resistance of microbial communities (Tylianakis and Morris, 2017); thus, we define the OTUs associated with these nodes as keystone species.


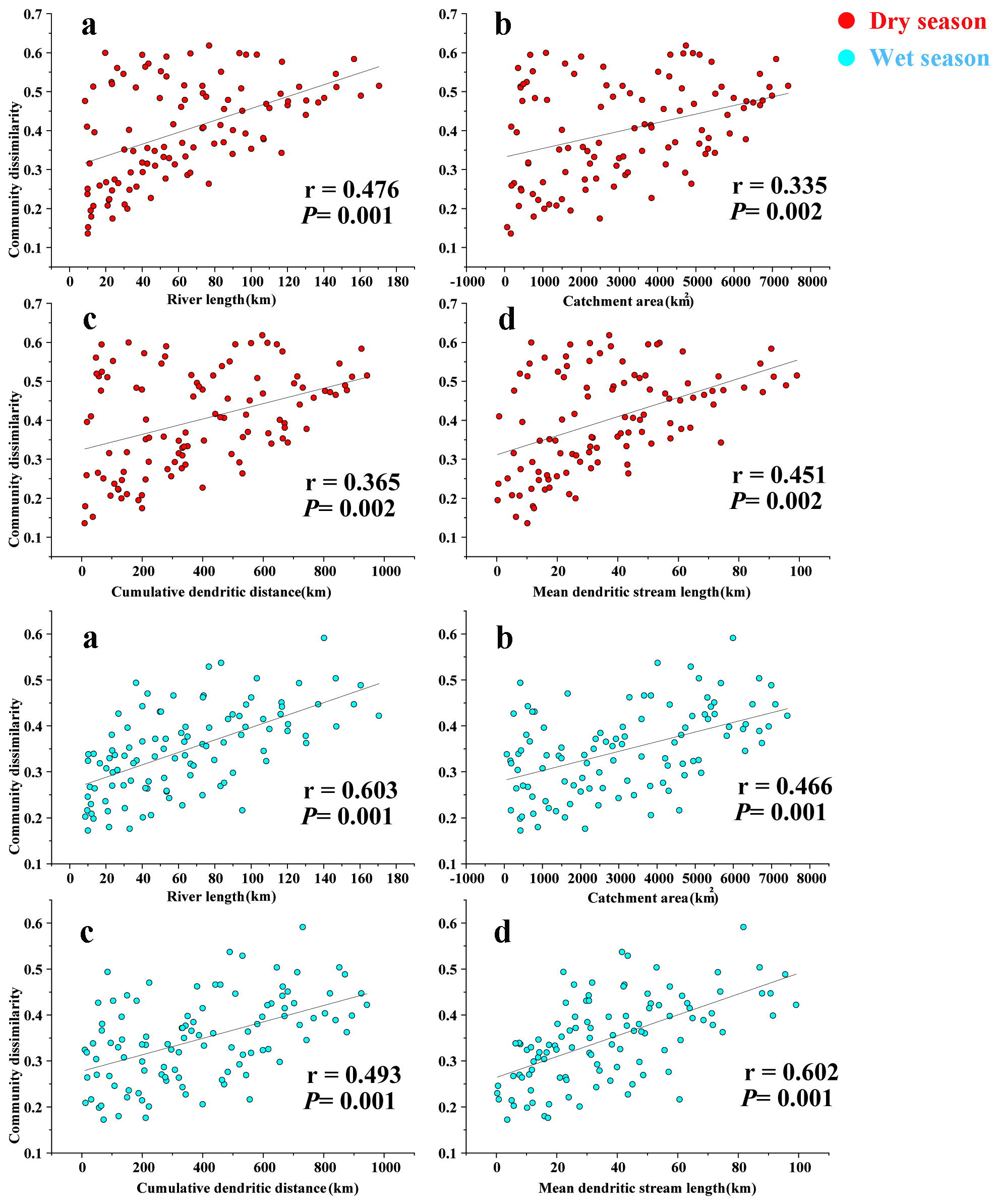


**FigureS8.** Bray–Curtis dissimilarity values of bacterioplankton communities in response to four geographic distance parameters. Relationships are shown between (a) river length (km), (b) catchment area (km^2^), (c) cumulative dendritic distance (km), and (d) mean dendritic stream length (km). Pearson correlations (r) and probabilities (*p*) are shown to the right of each plot.


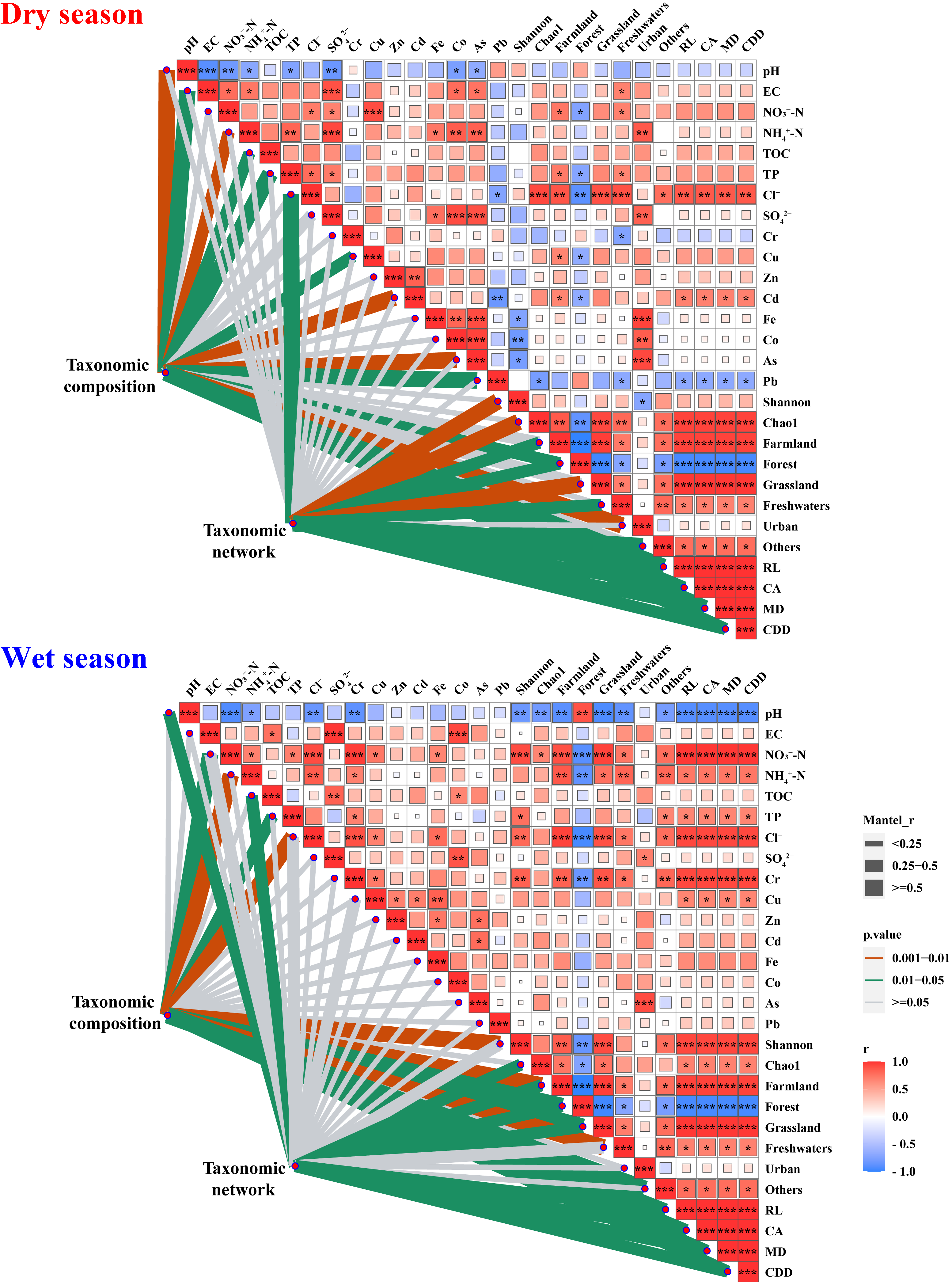


**FigureS9.** Mantel's correlations between environmental factors and the taxonomic compositions and networks of bacterioplankton communities in the Le'an River catchment. The widths of lines represent the magnitudes of Mantel's r statistic and the colors indicate the significance of a test; the sizes of colored blocks denote the significance of Pearson correlation between two environmental factors, and the absolute values of the Pearson correlation coefficient are labelled along the colored bar on the right.
